# Supplementary figures and images for: Provider performance and facility readiness for managing infections in young infants in primary care facilities in rural Bangladesh
Source: PLoS One. 2020 Apr 22;15(4):e0229988. doi: 10.1371/journal.pone.0229988 (PMC7176463; doi:10.1371/journal.pone.0229988)

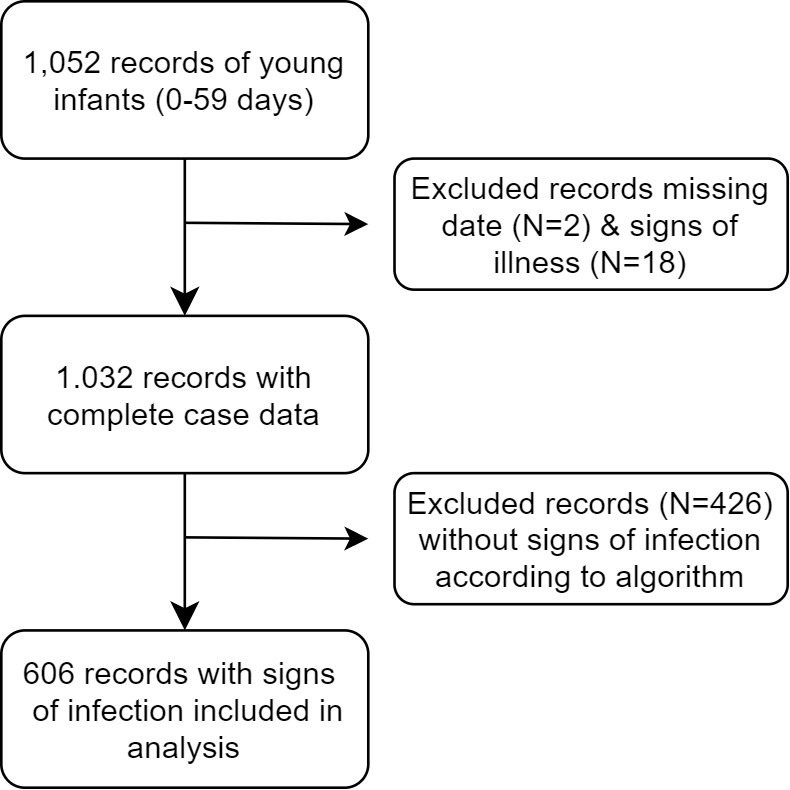

Supplement: S1 Fig — (TIF) [file pone.0229988.s004.tif]

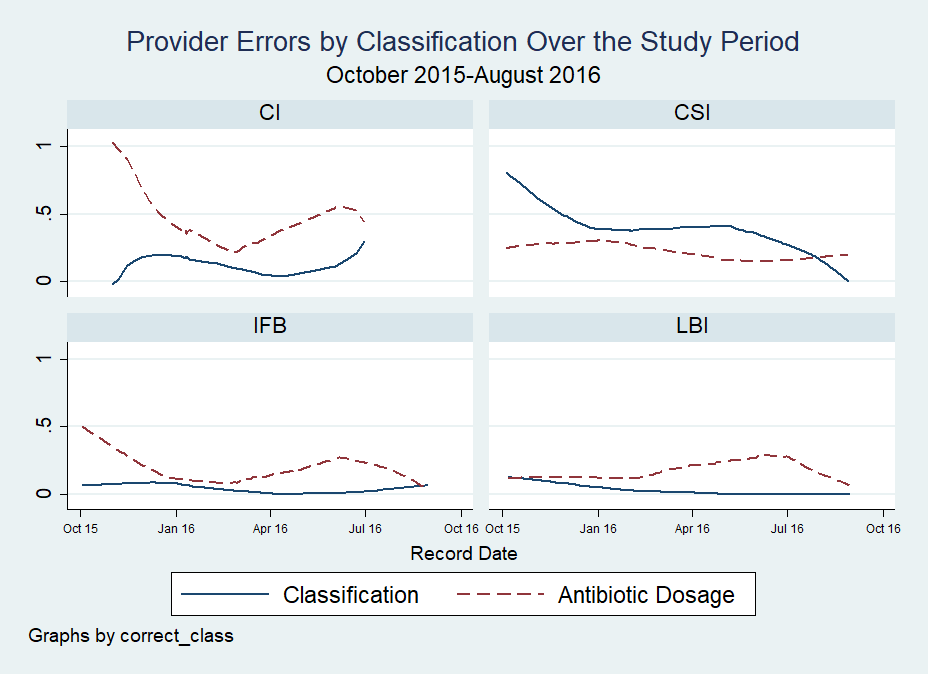

Supplement: S2 Fig — (TIF) [file pone.0229988.s005.tif]
