# Supplementary material for: Provider performance and facility readiness for managing infections in young infants in primary care facilities in rural Bangladesh
Source: PLoS One. 2020 Apr 22;15(4):e0229988. doi: 10.1371/journal.pone.0229988 (PMC7176463; doi:10.1371/journal.pone.0229988)
Supplement: Attachment — Submitted filename: My main recommendation.docx [file pone.0229988.s006.docx]

- My main recommendation is for the discussion. I feel it is not clear at the moment in the way it is laid out and I would re-arrange it to have a discussion on the results (main outcomes and rationale/comparison with other studies) followed by limitations and then recommendations.
- The conclusion in the abstract section does not match with the findings.
- Some acronyms should be spelled out at the first time written for instants GoB.
- Method section is not clear. If it is an interventional study, please follow the CONSORT guideline.
- Ethics: Add permit number.
- Why did not explained the agreement between the classification made by the provider and the computer algorism?
- Please add a flow diagram in a result section.
- In addition why did not calculate the sensitivity and specificity?
- It is better to have separate conclusion section.
